# Review of Non-Communicable Disease Research Activity in Kuwait: Where is the Evidence for the Best Practice?

**DOI:** 10.5334/aogh.2392

**Published:** 2019-03-21

**Authors:** Hanan Badr, Mohamad Ali Maktabi, Manal Al-Kandari, Abla M. Sibai

**Affiliations:** 1 University of Prince Edward Island (UPEI), CA; 2 American University of Beirut, LB; 3 Department of Community Medicine and Behavioral Sciences, Kuwait University, KW; 4 Department of Epidemiology and Population Health, American University of Beirut, LB

## Abstract

**Background:**

Kuwait, a small country in the Middle East, is now facing rapid development, with non-communicable diseases (NCDs) accounting for the majority of deaths.

**Objectives:**

In this study, we review trends in NCD research productivity in Kuwait and examine to what extent it is aligned with disease burden.

**Methods:**

Systematic mapping of NCD papers produced between January 2000 and December 2013 yielded 893 publications. These were defined according to study design, study focus, and risk factors examined. Research gaps were assessed by examining disparities between literature produced and cause-specific proportional mortality rates (PMR) and disability-adjusted life years (DALYs).

**Findings:**

While annual publication rates increased more than two-fold during the study period, many of the study methodologies were descriptive (58%). Only 2.6% were based on high-evidence interventional studies. Cancer, CVD, and diabetes featured in 38.1%, 15.1%, and 9.2% of the publications, respectively. Compared to PMR and DALYs, there was a surplus of cancer research, most of which were laboratory-based studies (27.6%) or of the case-report/case-series study type (26.5%). Smoking was more likely to be addressed in relation to CVD (32.6%) than diabetes (6.1%) or cancer (2.1%). Physical inactivity was mostly examined in its relation to diabetes (14.6%), with negligible representation in the remaining study focus (range 0.3% to 2.2%).

**Conclusion:**

NCD research production in Kuwait is not aligned with disease burden or health priorities. We recommend a coordinated action between funding agencies, universities, and researchers in Kuwait to guide development of a comprehensive research agenda that is responsive to the country’s emerging needs.

## Introduction

Non-communicable diseases (NCDs) encompass a broad range of conditions that together constitute a large and growing share of the disease burden worldwide. The Middle East and North Africa (MENA) region is now facing a fast rate of urbanization, with rates of NCDs increasing at an alarming rate. As early as 1997, the Global Burden of Disease Study identified NCDs as the leading causes of deaths in the Middle Eastern crescent, with subsequent evidence suggesting that the greatest increase is expected to occur in the region [1,2]. Brought about by modernization and economic development over the past few decades, Kuwait, a small high-income country of the MENA region, is undergoing a rapid epidemiologic transition characterized by a growing burden of NCDs, accounting for over 70% of total deaths [3]. It is an oil-producing country with a population of 4.7 million, the majority of whom are expatriates and migrant workers (70%). Despite concerted effort on behalf of the government, prevalence rates of NCD risk factors among nationals exceed those in several developed countries (e.g., 35% for current tobacco smoking among men; 63% for insufficient physical activity; with obesity rates among women exceeding 44%) [3,4].

Relative to other countries in the West, data for monitoring NCDs and their risk factors in Kuwait remain sparse, with little evidence that decisions and actions for addressing NCDs are grounded on evidence or have taken into account specific contextual factors and competing interests [5]. The mismatch between the health research that is needed and that which is conducted is a growing concern among scholars worldwide and in the region [6,7,8]. In their seminal paper on “Avoidable waste in the production and reporting of research evidence,” Chalmers and Glasziou [9] call for targeted research that addresses local concerns and priorities. Similarly, Rahim and colleagues [2] argue for the need to implement more research of relevance to the community and the importance of surveillance systems that assess interventions and monitor population-based initiatives.

In this study, we take a closer look at NCD research output in Kuwait for a better understanding of the knowledge produced and its potential in shaping programs and policies in the country. Using data from a large scoping review research, “Mapping of NCD Research in selected Arab Countries” in the MENA region [8], we aim in this study to review the landscape of NCD research produced in Kuwait from 2000 until 2013 and examine whether the output is aligned with disease burden in the country. Findings from this study have implications on priority-setting for NCD research agenda in Kuwait.

## Methods

### Search strategy and selection criteria

“Mapping of NCD Research in the Arab Region” is a four-year project funded by the International Development Research Centre (IDRC)-Canada (Ref 106981-00). This is a multi-country, multi-focal project that aims to screen NCD research output in the region, focusing on the World Health Organization (WHO) main categories of NCD and NCD risk factors. It seeks to identify the extent and scope of knowledge production, identify research gaps, overlaps and opportunities, and propose avenues that would support coordinated approaches for NCD research production in the region [8]. Due to the illustrative nature of the study purpose and research questions, the scoping systematic review methodology was carried out using Arksey and O’Malley framework [10]. Scoping studies are increasingly being implemented as evidence-mapping tools when the research question is broad and comprehensive in nature. The original NCD Research Mapping study explored NCD research production in seven selected Arab countries (Bahrain, Iraq, Kuwait, Lebanon, Morocco, Palestine, and Sudan) and spanned a 14-year period. The countries were selected to represent various stages of health transitions and economies in the region. This paper focuses on NCD research production from a single country, Kuwait. The detailed search strategy is provided elsewhere [8,11]. Briefly, we conducted an electronic search of the Medline database, accessed via PubMed, using both indexed terms and free text words for NCD and NCD risk factors. As such, we retrieved all relevant papers on NCD research in Kuwait that were published between January 2000 and December 2013. Papers were included in this study if their content focused on presentation, diagnosis, treatment, or care of any of the WHO’s four major NCD categories (cardiovascular disease [CVDs], cancer, diabetes mellitus [DM], and chronic respiratory diseases) or NCD risk factors; if the publication pertained to human health or health systems; and if the population under study consisted of Kuwaiti residents, including non-nationals. Title and abstract screening were conducted by two independent reviewers who examined the papers to see whether or not they fit the aforementioned criteria. Disagreement between any of the reviewers was resolved through discussion with the principal investigator. Initially, there were a total of 898 publications for Kuwait, of which 5 had no available full detailed information, yielding a final set of 893 unique publications.

### Data extraction and classification of key characteristics

Data pertaining to the journal identifiers (title, year of publication, and authors’ names and affiliations) were downloaded. Information on research participants (setting, targeted age groups), study design, and study focus were extracted from reading the abstract and full text. The study setting was differentiated based on the source of data, whether it was laboratory-based, hospital/clinic-based, community/population-based, or a special-interest group (e.g., registry of physicians). For studies involving human subjects, the targeted age groups were classified into children and adolescents, (<18 years), adults (between 18 and 50 years), older adults (>50 years), or other combinations of age groups. Study type/design was classified into case reports/case series, cross-sectional studies, case-control studies, cohort studies, intervention studies, reviews, laboratory studies (pathological or in-vivo studies), and others (e.g., qualitative studies, commentaries, and book chapters). Study focus was defined in terms of the main researched NCD and/or risk factor and research questions, as relevant. NCDs were considered in terms of the four main conditions based on the WHO classification, namely CVDs, cancer, DM, and chronic respiratory diseases. NCD risk factors comprised a long list of characteristics and behaviors; however, for the objectives of this study, we focused on four entities: tobacco, physical activity, dietary-related factors, and obesity. Data on authors’ collaboration between academic and other governmental or nongovernmental sectors were also recorded.

### Analyses

Data were transcribed into SPSS database (Version 20.1) for analyses. The distribution of NCD research output was plotted across the years of study, and a cross-tabulation was made between types of study design and focus of the research. The extent of research produced on each of the risk factors was estimated, stratified by study focus (four risk factors by four NCD outcomes). Finally, gap analysis was performed, examining disparities between the extent of NCD research produced and NCD burden in the country. The burden of NCD disease was expressed in terms of proportionate mortality rates (PMRs) from cancer, CVD, DM, and chronic respiratory diseases in the country [3], and in terms of disability-adjusted life year (DALYs) based on the Global Burden of Disease [12]. A benefit of the DALYs as a metric is that they integrate both mortality and morbidity measures.

## Results

**Figure 1:**
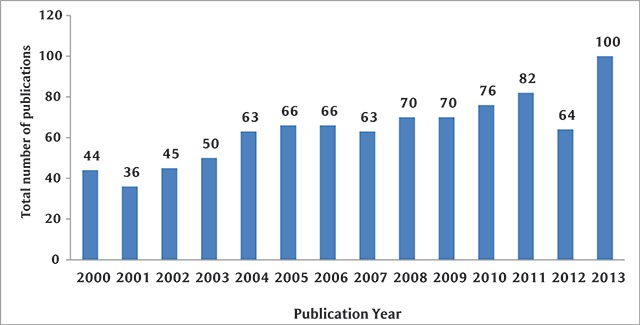
Trend in the annual number of NCD publications in Kuwait, 2000–2013.

The annual number of publications of NCD research in Kuwait increased consistently over the years, from 44 publications in 2000 to 100 in 2013, with a slight drop from the general trend occurring in 2012 (n = 64) (Figure 1). The most common study setting was the hospital/clinic (49.0%), followed by laboratory-based (22.5%) and community-based studies (12.0%) (data not shown). Out of 535 studies involving human subjects, 38.5% targeted adult participants aged between 18 and 50 years, 17.1% involved children and adolescents less than 18 years, and only 9.6% focused on older adults aged greater than 50 years. The rest of the studies (34.8%) included a combination of different age groups. Based on authors’ affiliations, most of the publications (61.9%) were carried out by academic researchers, notably from Kuwait University. An additional 20.3% of the studies were conducted in collaboration with the Ministry of Health in Kuwait, and 6.6% were led by the Kuwait Cancer Control Center (KCCC).

A large proportion of the publications focused on cancer studies (38.1%). This was followed by CVD studies (15.1%), DM (9.2%), and chronic respiratory diseases (6.2%) (Table 1). Over half (58.5%) were based on descriptive study designs (case reports/case series, cross-sectional study, case-control study, cohort study), with the majority using cross-sectional study designs. Interventional studies did not exceed 2.6%. Study designs varied significantly by type of NCD examined. Laboratory-based studies and case reports and case series were the most dominant study types for cancer studies (26.5%); cross-sectional study designs for chronic respiratory disease and DM (36.4% and 26.8%, respectively); and follow-up studies for CVDs (18.7%). Case-control study methodologies were equally employed for CVD, DM, and chronic respiratory disease (range 11.1%–15.9%). Review articles tackled cancer and CVDs (close to 13.5%) more than DM or chronic respiratory disease (7.3%–9.8%).

**Table 1:** Distribution of publications by study focus and study design, Kuwait, 2000–2013.

Study Focus	Study Type and Design																		

	Case Report/Case Series		Cross-Sectional Studies		Case-Control Studies		Cohort Studies		Intervention Studies		Review		Laboratory Studies		Others^b^		Total		P-Value

	n	%	n	%	n	%	n	%	n	%	n	%	n	%	n	%	N	%	

**Cancer**	90	26.5	40	11.8	17	5.0	37	10.9	12	3.5	46	13.5	94	27.6	4	1.2	340	38.1	<0.001
**Cardiovascular Disease**	8	6.0	25	18.5	15	11.1	25	18.5	4	3.0	18	13.3	34	25.2	6	4.4	135	15.1	0.008
**Diabetes Mellitus**	3	3.7	22	26.8	13	15.9	10	12.2	3	3.7	8	9.8	18	22.0	5	6.1	82	9.2	0.018
**Chronic Respiratory Disease**	2	3.6	20	36.4	7	12.7	3	5.5	2	3.6	4	7.3	15	27.3	2	3.6	55	6.2	0.136
**Others^a^**	14	5.0	117	41.6	28	10.0	26	9.3	2	0.7	22	7.8	66	23.5	6	2.1	281	31.4	0.463
**Total**	117	13.1	224	25.1	80	9.0	101	11.3	23	2.6	98	11.0	227	25.4	22	2.5	893	100.0	<0.001

^a^ Others include renal disease, palliative care, genetics, and other prevalence studies that examined NCD risk factors (behavioral and physiological). ^b^ Others include commentaries, qualitative studies, and book chapters.

**Figure 2:**
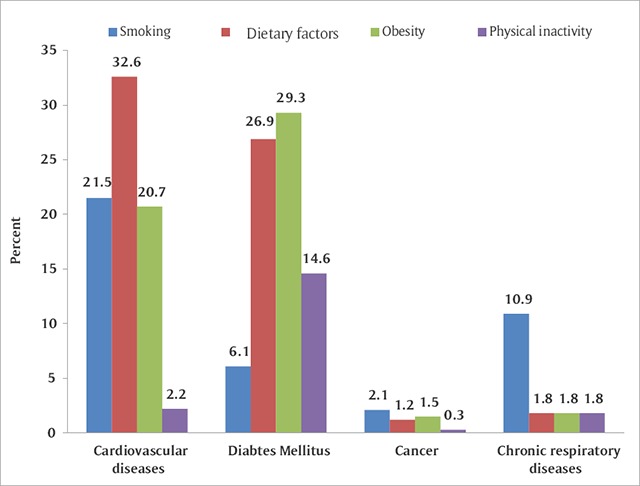
Distribution of risk factors addressed in NCD research, stratified by study focus,^a^ Kuwait, 2000–2013. ^a^ The combined percentages within each NCD focus do not necessarily total 100%, because papers may address more than one factor at a time or may address other factors not examined in the present analyses.

The extent to which each of the four risk factors (tobacco, physical activity, dietary-related factors, and obesity) were addressed across the four NCDs is illustrated in Figure 2. Smoking was mostly examined in publications focusing on CVDs (21.5%). This was followed, in descending order, by papers focusing on chronic respiratory diseases (10.9%) and DM (6.1%), and was least examined in cancer-related research (2.1%). Dietary factors were examined in 32.6% and 26.9% of the papers focusing on CVD and DM, respectively, while these were featured in only 1.2% of cancer-related studies. Obesity was examined in 29.3% and 20.7% of the studies dealing with DM and CVD, respectively. Finally, physical inactivity was mostly examined in association with DM (14.6%), with negligible representation in the remaining NCD studies (range 0.3% to 2.2%).

**Figure 3:**
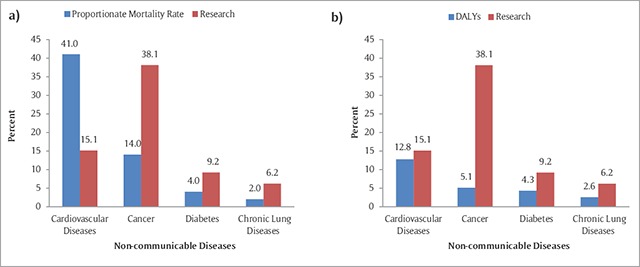
Gap analysis between NCD research output and NCD burden, expressed in proportionate mortality rates **(a)** and in DALYs **(b)**, Kuwait, 2000–2013.

Figure 3 presents the findings of the gap analyses and attempts to answer the question whether Kuwait is producing NCD research proportional to its country-specific breakdown of NCD burden, based on cause-specific PMRs and DALYs for the four major groups of NCDs (CVD, cancers, DM, and chronic respiratory diseases). Compared to disease burden of NCD, results showed a clear relative surplus of research produced on cancer.

## Discussion

The need for a stronger scientific evidence base for curbing the NCD epidemic has long been identified by public health actors and various stakeholders. Concurrently, it is crucial to ensure that data and research output match national disease burden and public health priorities, such that countries worst affected by NCDs are producing a considerable amount of literature for monitoring and evaluation. The present study showed that Kuwait has been overall increasingly productive in NCD research, with more than a two-fold increase in output from 2000 until 2013. The slight drop around 2012 has been observed in other reviews in the region and is likely to be the result of the political upheavals that struck the region in 2011 and were at their peak in 2012 [13]. The rise in publication rate over time is higher than the trend in other countries in the region but remains lower than, for example, Lebanon, a middle-income country, where the increase has been close to six-fold [8]. It is worth noting that Kuwait has so far conducted two STEPwise surveys, in 2006 [14] and 2013 [4], yet the output from this effort was limited to two country reports and merely one paper in a refereed journal [15]. In future studies, research output from Kuwait need to be appraised vis-à-vis resources and policies at the individual (e.g., quantity and quality of researchers), institutional (university promotion incentives and guidelines, library resources and equipment, funding opportunities), and governmental levels (GDP, research and development spending).

Findings from this review showed that CVD studies occupied only 15% of the NCD research in Kuwait during the study period. While this fell behind its cause-specific burden assessed in terms of PMRs, the deficit in CVD research output was diluted and became negligible when the burden was assessed in terms of DALYs. This is because DALYs take into consideration both mortality and morbidity characteristics of the disease, and compared to cancer, for example, CVD has lower case fatality rates. The majority of the CVD studies were population-based, with cross-sectional and follow-up studies constituting half of the research produced in this subject area. Hence, risk factor epidemiology topped the focus of CVD research, with smoking, dietary factors, and obesity being addressed in a substantial proportion of the studies. Yet, the proportion of studies that examined associations with physical activity was negligible (2.2%). Except for diabetes, this deficiency was observed across NCD groups. Given the high prevalence of physical inactivity in Kuwait, it is worrisome to note the low interest among researchers in studying physical inactivity in the country. Today, lack of physical inactivity is the fourth leading risk factor for global mortality and is one of the leading public health indicators [16]. Physical activity in many countries of the Gulf region is hampered by lack of public parks, reduced opportunities for exercise, and the hot climate and car-dominated transport. Also, women are less likely to be members of the workforce, further restricting their potentiality for a physically active lifestyle. These structural determinants with context-specific research questions need to be addressed in future studies.

Compared to other NCD disease groups, cancer studies topped the list of NCD literature in Kuwait (38.1%), with findings suggesting a surplus in cancer research when compared to disease burden, whether expressed as PMRs or DALYs (Figure 3). Furthermore, the majority of studies were accounted for by laboratory-based studies and lower-evidence research (e.g., case reports/case series) that lacked the epidemiological research framework needed to address analytical questions and etiologies. This explains our finding of the relatively low percentages of cancer studies that focus on risk factors (range 0.3% to 2.1%). Cancer accounts for 10 of the top 25 causes of death in Kuwait [3], and cancer-related behavioral risk factors are highly prevalent in the Kuwaiti adult population. For instance, the Kuwait STEPs 2014 survey revealed that, among adults aged 18–69, the prevalence of smoking among men is 39.2%, overweight is 77.2%, and inadequate diet is 83.8% [4]. With such a high prevalence of cancer-related risk factors in the country, it is worrisome to note in our study the low interest of risk-factor epidemiology in cancer research. Etiology and epidemiological studies are of relevance not only to the scientific community but also to policy makers and program developers, for a better understanding of the attributable risk to well-known risk factors and for monitoring progress in the country.

Kuwait is known to host one of the highest prevalence rates of DM worldwide. With a prevalence of 15.8% among adults aged between 20 and 79, Kuwait ranks in the top eighth percentile worldwide [17]. Yet, our gap analysis shows that diabetes has remained a subordinate topic of interest to researchers, occupying less than 10% of the research produced on NCD during the study period. Although the national Dasman Diabetes Institute, established in 2006, has been playing a leading role in combatting the diabetes epidemic in Kuwait through focused diabetes research, integrated prevention, training, and education, our findings are compelling for the need to promote more investment in community genetic research targeting NCDs with the highest morbidity, heritability, and health burden [11].

This review identified other issues that are worth noting. A small proportion of the studies targeted adults greater than 50 years of age. The lack of representation of the older population has been observed in an earlier review of cancer publications in the region [18] and is a deficiency that needs to be addressed in future studies. Moreover, almost half of the studies were conducted in a hospital or clinical setting, with few being population-based with a public health perspective for prevention and control. Also, it was particularly disturbing to note the low proportion of publications that featured high-evidence research, notably intervention studies (less than 3%). On the other hand, while most of the studies were conducted by the Kuwait University, the only public academic institution in the country, the engagement of the Ministry of Health in the production of knowledge on NCD is noteworthy. This can be seen as conducive to raising awareness among policy makers, healthcare providers, and other stakeholders of the vital need for evidence to guide the planning of interventions and programs.

One of the limitations of this review is that it is based on a single search engine. Also, the analyses covered only scientific publications in peer-reviewed journals, thus missing further evidence from the grey literature or other reports that remain unpublished. Yet, this remains the first detailed analyses of NCD research productivity in Kuwait providing a case study for mapping NCD research production in other countries in the region and elsewhere.

Health coverage and access to care, including cost of chronic medications, is free for Kuwaiti citizens. This is not the case for migrant workers, who have to pay for health insurance and for some services, such as laboratory and radiological investigations [19]. This may adversely affect compliance rates among expatriates and result in worsening of NCD burden. Yet, Kuwait has a medical informatics system which can track an individual’s interaction with the healthcare system that is well implemented in primary care services. A working plan is underway to extend this service to the secondary and tertiary health services, which is expected to be in service soon. This will facilitate outcome research with a better assessment of the evidence-based and guideline-concordant performance of NCD care in Kuwait.

In conclusion, findings from this study reveal three main areas of concern. Firstly, there is a surplus of cancer studies—and these are mostly of the case-report or case-series type, which lack a methodological framework for examining associations with risk factors and assessing attributable risks. Secondly, despite the huge burden of DM in Kuwait, this remains an under-researched topic. Thirdly, there is an overall deficiency of studies that address physical activity. This is a missed opportunity for greater awareness and recognition by health professionals and policy makers of the importance of physical activity for NCD prevention and control. NCD is a global health issue, with recent calls for research that would inform policy reforms. Similarly, there have been international calls for revising funding flows and improving research stewardship for “reducing research waste and increasing value.” There is a misperception that this may not be relevant to high-income countries. This study calls for a coordinated discussion and a think tank comprising research centers, universities, and researchers in Kuwait that would support a better understanding of the NCD research landscape and approaches towards shaping NCD research agenda priorities responsive to emerging needs in the country. We hope that findings from this study will incite further discussions on research priority setting and funding allocation in the region and beyond.

## Abbreviations

CVD: Cardiovascular disease; DALY: Disability-adjusted life year; DM: Diabetes mellitus; IDRC: International Development Research Centre; KCCC: Kuwait Cancer Control Center; MENA: Middle East and North Africa; NCDs: Non-communicable diseases; PMR: Proportional mortality rate; WHO: World Health Organization

## References

[1] Murray C and Lopez A. Mortality by cause for eight regions of the world: Global Burden of Disease Study. The Lancet. 1997; 349(9061): 1269–1276. DOI: 10.1016/S0140-6736(96)07493-4 9142060

[2] Rahim H, Sibai AM, Khader Y, et al. Non-communicable diseases in the Arab world. The Lancet. 2014; 383(9914): 356–367. DOI: 10.1016/S0140-6736(13)62383-1 24452044

[3] World Health Organization. Noncommunicable Diseases (NCD) Country Profiles. WHO.int. 2014 http://www.who.int/nmh/countries/kwt_en.pdf?ua=1. Accessed February 3, 2018.

[4] Ministry of Health in Kuwait, Eastern Mediterranean Approach for Control of Non Communicable diseases (EMAN). Survey for risk factors for chronic non-communicable diseases. WHO.int. 2015 http://www.who.int/chp/steps/Kuwait_2014_STEPS_Report.pdf?ua=1. Accessed December 10, 2017.

[5] World Health Organization. A prioritized research agenda for prevention and control of NCDs: CVD, cancer, chronic respiratory disease, diabetes. WHO.int. 2011 http://www.who.int/cardiovascular_diseases/publications/ncd_agenda2011/en/. Accessed January 7, 2017.

[6] Røttingen J, Regmi S, Eide M, et al. Mapping of available health research and development data: what’s there, what’s missing, and what role is there for a global observatory? The Lancet. 2013; 382(9900): 1286–1307. DOI: 10.1016/S0140-6736(13)61046-6 23697824

[7] Albarqouni L, Elessi K and Abu-Rmeileh N. A comparison between health research output and burden of disease in Arab countries: evidence from Palestine. Health Research Policy and Systems. 2018; 16(1): 25 DOI: 10.1186/s12961-018-0302-4 29544498 PMC5856204

[8] Sibai AM, Singh N, Jabbour S, et al. Does published research on non-communicable disease (NCD) in Arab countries reflect NCD disease burden? PLOS ONE. 2017; 12(6): e0178401 DOI: 10.1371/journal.pone.0178401 28575065 PMC5456081

[9] Chalmers I and Glasziou P. Avoidable waste in the production and reporting of research evidence. The Lancet. 2009; 374(9683): 86–89. DOI: 10.1016/S0140-6736(09)60329-9 19525005

[10] Arksey H and O’Malley L. Scoping studies: Towards a methodological framework. International Journal of Social Research Methodology. 2005; 8(1): 19–32. DOI: 10.1080/1364557032000119616

[11] Jamaluddine Z, Sibai AM, Othman S and Yazbek S. Mapping genetic research in non-communicable disease publications in selected Arab countries: first step towards a guided research agenda. Health Research Policy and Systems. 2016; 14(1). DOI: 10.1186/s12961-016-0153-9 PMC5103400 27832776

[12] Institute for Health Metrics and Evaluation. GBD Compar IHME Viz Hub. Vizhub.healthdata.org. 2016 http://vizhub.healthdata.org/gbd-compare/ 1 2, 2017.

[13] Makhoul J, Chehab R, Shaito Z and Sibai A. A scoping review of reporting “Ethical Research Practices” in research conducted among refugees and war-affected populations in the Arab world. BMC Medical Ethics. 2018; 19(1). DOI: 10.1186/s12910-018-0277-2 PMC5952584 29764456

[14] Ministry of Health in Kuwait, Eastern Mediterranean Approach for Control of Non-Communicable diseases (EMAN). Survey for risk factors for chronic non-communicable diseases. ISBN 978-99906-613-3-0 WHO.int. 2008/090. http://www.who.int/chp/steps/STEPS_Report_Kuwait.pdf. Accessed December 10, 2017.

[15] Alarouj M, Bennakhi A, Alnesef Y, Sharifi M and Elkum N. Diabetes and associated cardiovascular risk factors in the State of Kuwait: the first national survey. International Journal of Clinical Practice. 2012; 67(1): 89–96. DOI: 10.1111/ijcp.12064 23241053

[16] Kohl H, Craig C, Lambert E, et al. The pandemic of physical inactivity: global action for public health. The Lancet. 2012; 380(9838): 294–305. DOI: 10.1016/S0140-6736(12)60898-8 22818941

[17] International Diabetes Federation. Countries ranked by diabetes prevalence (% of population ages 20 to 79). Indexmundi.com. 2015 https://www.indexmundi.com/facts/indicators/SH.STA.DIAB.ZS/rankings. Accessed January 17, 2018.

[18] Hamadeh R, Borgan S and Sibai AM. Cancer Research in the Arab world: A review of publications from seven countries between 2000ge>–2013. Sultan Qaboos University Medical Journal. 2017; e147–154. DOI: 10.18295/squmj.2016.17.02.003 PMC5488814 28690885

[19] The Public Authority for Civil Information (PACI). Statistics Services System. Kuwait. https://www.paci.gov.kw/stat/. Accessed January 18, 2019.

